# Agreement of wall shear stress distribution between two core laboratories using three-dimensional quantitative coronary angiography

**DOI:** 10.1007/s10554-023-02872-4

**Published:** 2023-05-27

**Authors:** Shigetaka Kageyama, Vincenzo Tufaro, Ryo Torii, Grigoris V. Karamasis, Roby D. Rakhit, Eric K. W. Poon, Jean-Paul Aben, Andreas Baumbach, Patrick W. Serruys, Yoshinobu Onuma, Christos V. Bourantas

**Affiliations:** 1 Department of Cardiology, University of Galway, College of Medicine, Nursing and Health Sciences, Galway, Ireland; 2 Department of Cardiology, Barts Heart Centre, Barts Health NHS Trust, West Smithfield, London, EC1A 7BE UK; 3 Centre for Cardiovascular Medicine and Devices, William Harvey Research Institute, Queen Mary University of London, London, UK; 4 Department of Biomedical Sciences, Humanitas University, Pieve Emanuele-Milan, Italy; 5 Department of Mechanical Engineering, University College London, London, UK; 6 Essex Cardiothoracic Centre, Basildon, UK; 7 Royal Free Hospital, London, UK; 8 Department of Mechanical Engineering, Melbourne School of Engineering, The University of Melbourne, Melbourne, Australia; 9 Pie Medical Imaging, Maastricht, The Netherlands; 10 National Heart and Lung Institute, Imperial College London, London, UK; 11 Institute of Cardiovascular Sciences, University College London, London, UK; 12 Department of Cartiology, Galway University Hospitals, Galway, Ireland

**Keywords:** Computational fluid dynamics, Quantitative coronary angiography, Reproducibility, Wall shear stress

## Abstract

Wall shear stress (WSS) estimated in models reconstructed from intravascular imaging and 3-dimensional-quantitative coronary angiography (3D-QCA) data provides important prognostic information and enables identification of high-risk lesions. However, these analyses are time-consuming and require expertise, limiting WSS adoption in clinical practice. Recently, a novel software has been developed for real-time computation of time-averaged WSS (TAWSS) and multidirectional WSS distribution. This study aims to examine its inter-corelab reproducibility. Sixty lesions (20 coronary bifurcations) with a borderline negative fractional flow reserve were processed using the CAAS Workstation WSS prototype to estimate WSS and multi-directional WSS values. Analysis was performed by two corelabs and their estimations for the WSS in 3 mm segments across each reconstructed vessel was extracted and compared. In total 700 segments (256 located in bifurcated vessels) were included in the analysis. A high intra-class correlation was noted for all the 3D-QCA and TAWSS metrics between the estimations of the two corelabs irrespective of the presence (range: 0.90–0.92) or absence (range: 0.89–0.90) of a coronary bifurcation, while the ICC was good-moderate for the multidirectional WSS (range: 0.72–0.86). Lesion level analysis demonstrated a high agreement of the two corelabls for detecting lesions exposed to an unfavourable haemodynamic environment (WSS > 8.24 Pa, κ = 0.77) that had a high-risk morphology (area stenosis > 61.3%, κ = 0.71) and were prone to progress and cause events. The CAAS Workstation WSS enables reproducible 3D-QCA reconstruction and computation of WSS metrics. Further research is needed to explore its value in detecting high-risk lesions.

**Supplementary Information:**

The online version contains supplementary material available at 10.1007/s10554-023-02872-4.

## Introduction

Wall shear stress (WSS) in human coronary arteries has a major impact on endothelial function and plays a key role in atherosclerotic disease development, plaque destabilization and rupture.

Several studies have shown that WSS computations enable the identification of high-risk lesions [1, 2]; however, despite this compelling evidence its clinical applications are currently limited. This should be attributed to the facts that WSS computation requires accurate vessel reconstruction—traditionally performed using fusion of intravascular imaging and angiographic data—and that the processing of the generated geometries using computational fluid dynamics (CFD) techniques is laborious, time-consuming and requires expertise [3–6].

To overcome the limitations of conventional CFD-based analysis, WSS calculation software solely based on three-dimensional quantitative coronary angiography (3D-QCA) was developed (CAAS Workstation WSS prototype software, Pie Medical Imaging, Maastricht, the Netherlands). The software allows fast 3D-QCA reconstruction and WSS computation on a standard computer within a short analysis time of minutes [7]. Previous studies have demonstrated that 3D-QCA derived WSS using processing of the 3D reconstructed coronary geometries with CFD techniques enables accurate quantification of flow patterns and WSS distribution [8] and potentially detection of lesions causing major adverse cardiovascular events (MACE) [9–11]; in addition, a recent study has shown a high agreement between the estimations of the CAAS Workstation WSS software and the WSS computed using conventional CFD analysis in 3D-QCA reconstructions [7].

Since WSS calculation involves multiple steps of analysis, it is important to evaluate the reproducibility of WSS computation using the CAAS Workstation WSS software between two imaging core laboratories. [12] The aim of this study is therefore to present inter-corelab reproducibility of WSS using this novel software.

## Methods

### Studied patients

In this analysis, 60 cases were selected from a multicenter registry of patients who had at least one non-flow limiting lesion with borderline negative fractional flow reserve (FFR: 0.81–0.85). [10] The angiograms of 40 lesions without any side branch > 1.0 mm diameter and 20 lesions with 1 major side branch were included in the present analysis.

This study was conducted as part of a local audit to assess the outcome of patients with borderline negative FFR lesions treated conservatively following current international guidelines. [13] All patient-identifiable information was removed before transferring data to the corelabs. The local committee advised that formal ethical approval was not required for this retrospective study.

### Imaging transfer and preparation of analysis in 2 core labs

Coronary angiographies were anonymized and transferred to two corelabs (University of Galway Corrib Core Lab, Galway, Ireland and Barts Heart Centre, London, United Kingdom) in digital imaging and communications in medicine (DICOM) format. The two core labs used the same angiographic projections and the same frame for 3D coronary reconstruction as well as the same side branches to define the segment of interest. The two corelabs were blinded to the clinical information of the selected patients and lesions including FFR values. Both corelabs performed the analysis according to the standard of operation of each institution.

### 3D-QCA reconstruction and WSS computation

**Fig. 1:**
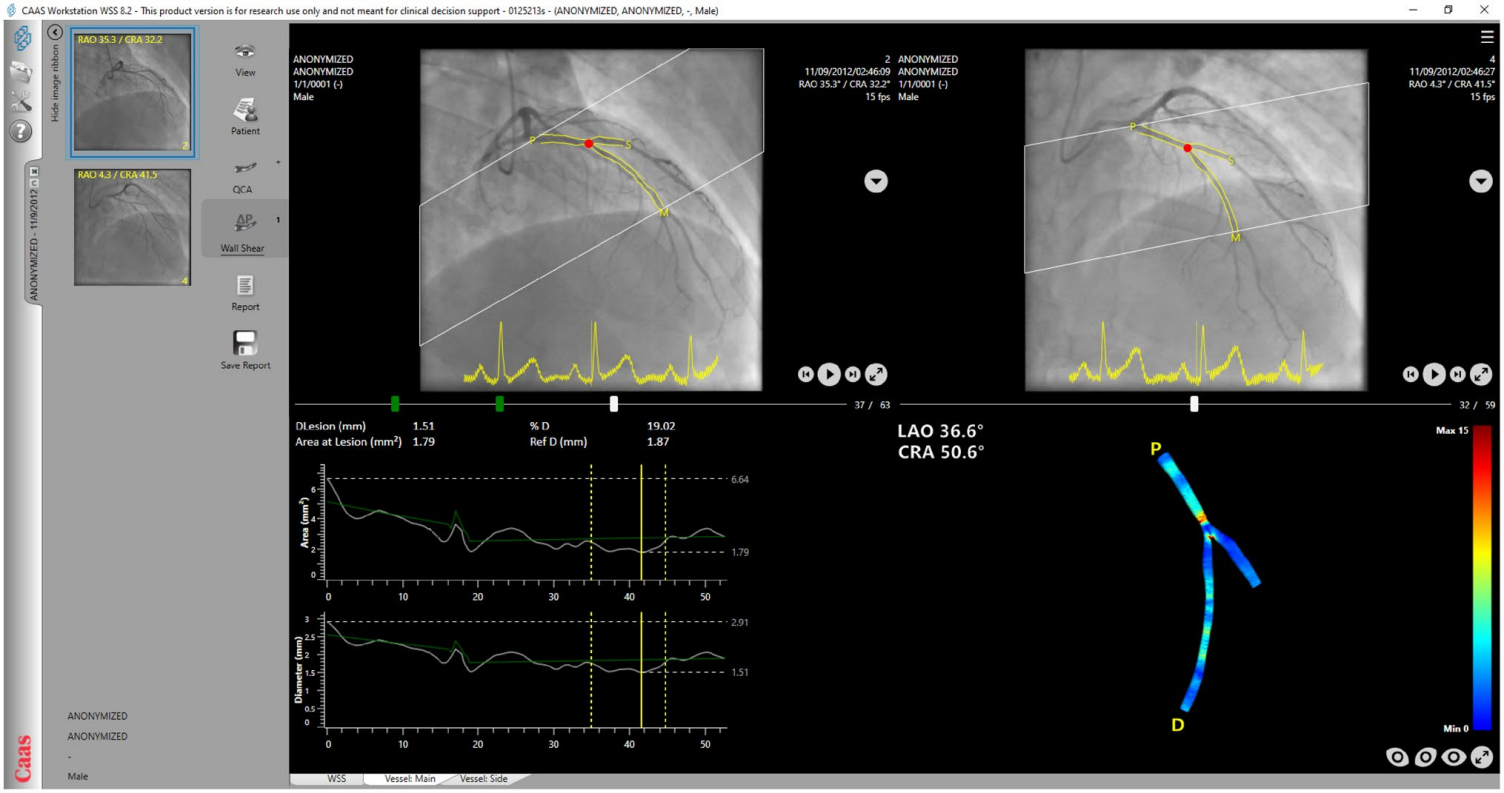
CAAS Workstation WSS software allowing real-time 3D-QCA analysis and WSS computation

Side branches located proximally and distally to the studied lesion were used as fiducial landmarks to define a segment of interest; 3D-QCA reconstruction of the segment of interest was performed from two end-diastolic angiographic projections at least 30° apart using the CAAS workstation WSS software (Fig. 1). An edge detection algorithm was used to detect the lumen borders in the two end-diastolic projections; manual corrections were performed if these were deemed necessary. The detected lumen borders were used to generate elliptical cross-sections of the lumen that constituted the 3D geometry of the segment of interest.

Model meshing was performed by the software using tetrahedral elements applying a curvature-based approach that determines elements size based on vessel curvature (small element size in curved and large in straight segments). Near the wall, the mesh had a prismatic 3 layer of elements. The curved-based approach and small number of near-wall layers enable fast analysis without compromising WSS computations [7].

A pulsatile flow profile was applied at the inlet of the reconstructed segment based on generic time-varying Doppler velocity curves of the studied vessel (e.i, left anterior descending artery, left circumflex or right coronary artery). [11] The blood flow velocity at the inflow of the model was patient-specific and estimated from the model length, the frame rate of the analysed angiogram and the number of frames required for the contrast to fill the reconstructed segment. [10, 14] The same boundary conditions were imposed in the models reconstructed by the two corelabs. More specifically, blood was assumed to be a Newtonian fluid with a density of 1060 kg/m^3^ and viscosity of 0.0035 Pa s, while the flow was assumed to be homogeneous and incompressible. The wall of the 3D model was considered to be rigid, no-slip conditions were applied at the wall boundaries, while zero pressure conditions were imposed at the outflow of the model. In models with a side branch, the empirical model proposed by Giessen et al. was applied to estimate the flow split between the two branches [15].

The governing equations of fluid motion were solved by the CAAS Workstation WSS software in their discretized form under transient-state conditions by applying the finite element code Kratos [15] which is specially designed to perform CFD analysis in coronary arteries.

### Data extraction and statistical analysis

To examine the reproducibility of the software in assessing model geometry and the distribution of the local haemodynamic forces the following variables were estimated and compared between the two core labs:

1. 3D-QCA metrics including the model and lesion length, mean reference lumen area, minimum lumen area (MLA) and % area stenosis (AS) defined by the following equation:
  $$\% {\text{AS}} = \frac{{{\text{Reference lumen area at the MLA}}\, - \,{\text{MLA}}}}{{{\text{Reference lumen area at the MLA}}}}\, \times \,100$$

The reference area at the MLA was estimated from the proximal and distal reference lumen area using a linear interpolation approach. In addition, model length—in the bifurcation model, length of proximal segment, main branch after bifurcation, and side branch after bifurcation and bifurcation angle—were computed and compared in order to examine the anatomical agreements of the reconstructed models.

2. The reconstructed segment was split into 3 mm segments and for each segment, the mean time-averaged (TA) WSS, the maximum predominant and the minimum predominant TAWSS values were estimated for the entire reconstruction as previously described. [1, 2, 9] Predominant maximum and minimum TAWSS were defined as the maximum and minimum values of the mean TAWSS of all surface data points located in a 90° arc window in a 3 mm segment across the circumference of the vessel wall. [2] In the case of bifurcations, only the TAWSS distribution in the proximal vessel and its main branch were extracted [17]. Moreover, 4 types of multidirectional WSS were computed and compared in each 3 mm segment: the maximum predominant oscillatory index (OSI) that reflects the changes in the direction and magnitude of the shear stress, the relevant residence time (RRT) that indicates the time that blood particles reside at a certain location at the vessel wall, the maximum predominant transverse wall shear stress (transWSS) which indicates the shear stress component that is perpendicular to the mean WSS vector during the cardiac cycle, and the maximum predominant cross-flow index (CFI) which is the transWSS normalized by the TAWSS. The mathematical formulas describing these metrics are shown in the Supplementary Table 1. [18, 19]
3. In addition, the TAWSS and multidirectional WSS distribution in the segments at the point of bifurcation (POB) [20] were compared in bifurcation lesions.
4. Finally, for each lesion the following WSS values were computed: the maximum value among the mean TAWSS value computed in the 3 mm segments that consist of the lesion, the maximum value amongst the maximum predominant TAWSS estimated in the 3 mm segments of the lesion, and the minimum value amongst the minimum predominant TAWSS estimated in the 3 mm segments of the lesion. These values corresponded to the maximum TAWSS, the maximum predominant TAWSS and the minimum predominant TAWSS of the lesion respectively.

Continuous variables were presented as mean SD, while categorical variables as absolute numbers and percentages. Comparison of the estimations of the two corelabs was performed using Wilcoxon signed rank test. Intra-class correlation coefficient (ICC) with p values for a null hypothesis as ICC = 0 and Bland-Altman analysis were also used to investigate the agreement between the estimations derived by the two approaches.

We have recently reported that lesions with an AS > 61.3% and maximum TAWSS > 8.24 Pa are likely to progress and cause events. [10] To examine the efficacy of the two corelabs in identifying lesions with unfavourable anatomy (%AS > 61.3) exposed to a high-risk haemodynamic environment (maximum TAWSS > 8.24 Pa) we plotted these variables and divided them into 4 quadrants using the 61.3% for the AS and the 8.24 Pa for the TAWSS as cutoffs. The agreement of the two core labs was tested using Cohen’s Kappa index.

All statistical analyses were performed using R 4.1.1 (The R foundation for statistical computing, Vienna, Austria). All reported P-values were two-sided, and P < 0.05 was considered statistically significant.

## Results

### Baseline characteristics

Sixty patients with 60 lesions were included in the current study. The mean age of the patients was 63.9 ± 10.6 years. More than half of the patients were suffering from hypertension and dyslipidemia, while the incidence of diabetes was 30.5%; 86.7% of the patients underwent a coronary angiogram because of a chronic coronary syndrome and 13.3% because of an admission with a non-ST elevation myocardial infarction—in the latter population, the non-culprit lesions were assessed by FFR. The baseline characteristics of the studied patients and lesions are shown in Table 1.

**Table 1:** Baseline demographics of the studied patients

	Studied patients (n = 60)	Straight model (n = 40)	Bifurcation model (n = 20)
Age (years)	63.9 ± 10.6	63.7 ± 11.2	64.0 ± 9.4
Male	51 (85.0%)	32 (80.0%)	19 (95.0%)
Clinical presentation			
Chronic coronary syndrome	52 (86.7%)	35 (87.5%)	17 (85.0%)
Acute coronary syndrome	8 (13.3%)	5 (12.5%)	3 (15.0%)
Co-morbidities			
Hypertension	37 (63.8%)	26 (68.4%)	11 (55.0%)
Hypercholesterolemia	33 (55.0%)	22 (55.0%)	11 (55.0%)
Diabetes mellitus	18 (30.5%)	13 (33.3%)	5 (25.0%)
History of smoking^a^	33 (55.0%)	23 (57.5%)	10 (50.0%)
Reduced LVEF^b^	6 (10.3%)	2 (5.1%)	4 (21.1%)
CKD^c^	11 (18.6%)	8 (20.5%)	3 (15.0%)
Previous MI	12 (20.0%)	8 (20.0%)	4 (20.0%)
Previous PCI	22 (36.7%)	16 (40.0%)	6 (30.0%)
Lesion location			
Right coronary artery	16 (26.6%)	16 (40.0%)	0 (0%)
Left anterior descending	38 (63.4%)	19 (47.5%)	18 (95.0%)
Left circumflex	6 (10.0%)	5 (12.5%)	1 (5.0%)
Fractional flow reserve (FFR)			
FFR	0.83 ± 0.01	0.83 ± 0.01	0.84 ± 0.01

*ACE* angiotensin-converting enzyme, *ACS* acute coronary syndrome, *CAD* coronary artery disease, *CAG* coronary angiography, *CKD* chronic kidney disease, *LVEF* left ventricular ejection fraction, *MI* myocardial infarction, *PCI* percutaneous coronary intervention; fractional flow reserve

^a^History of smoking, defined as current or previous smoker

^b^Reduced LVEF, defined as ejection fraction < 50%

^c^CKD, defined as estimated glomerular filtration rate < 60mL min^−1^ 1.73m^2^

### Inter-core lab agreement of 3D-QCA parameters

A high inter-corelab agreement was observed in the absolute measurements of luminal dimensions (model length, lesion length, and MLA) with excellent ICC ≥ 0.92 in both non-bifurcated and bifurcated models, whereas the ICC for the % AS was 0.88 and 0.90, respectively (Table 2). Bland-Altman analysis also confirmed a small bias and narrow limits of agreement between the two corelabs for all the above metrics (Fig. 1s).

**Table 2:** Comparison of the 3D QCA components between two corelabs

	1st corelab estimations	2nd corelab estimations	Mean ± SD of the differences	P value (paired)	ICC	P value (ICC)
*Non-bifurcated models (n = 40)*						
Model length (mm)	41.80 ± 16.27	41.62 ± 16.12	0.18 ± 1.43	0.106	0.99 (0.99–1)	< 0.001
Lesion length (mm)	16.14 ± 6.39	16.09 ± 7.32	0.05 ± 2.76	0.428	0.92 (0.86–0.96)	< 0.001
MLA (mm^2^)	2.69 ± 1.13	2.63 ± 1.11	0.06 ± 0.36	0.13	0.95 (0.91–0.97)	< 0.001
Area stenosis (%)	52.31 ± 9.93	53.06 ± 10.70	− 0.74 ± 5.14	0.376	0.88 (0.79–0.93)	< 0.001
*Bifurcated models (n = 20)*						
Proximal MB (mm)	11.47 ± 6.26	11.53 ± 6.12	− 0.05 ± 0.72	0.57	0.99 (0.98–1)	< 0.001
Distal MV (mm)	23.78 ± 7.52	24.23 ± 7.41	− 0.45 ± 0.92	0.13	0.98 (0.98–1)	< 0.001
Distal SB (mm)	12.24 ± 3.55	12.30 ± 3.71	− 0.06 ± 0.64	0.705	0.99 (0.97–0.99	< 0.001
Angle (°)	59.06 ± 13.68	57.51 ± 14.56	1.54 ± 4.65	0.312	0.95 (0.87–0.98)	< 0.001
Lesion length (mm)	10.44 ± 3.77	10.98 ± 4.22	− 0.54 ± 1.21	0.059	0.95 (0.86–0.98)	< 0.001
MLA (mm^2^)	2.09 ± 0.73	2.08 ± 0.77	0.004 ± 0.13	0.763	0.99 (0.97–0.99)	< 0.001
Area stenosis (%)	45.43 ± 12.76	45.49 ± 13.24	− 0.06 ± 6.16	0.841	0.90 (0.77–0.96)	< 0.001

*3D* three-dimensional, *QCA* quantitative coronary angiography, *ICC* interclass correlation coefficient, *MLA* minimum lumen area, *MB* main branch, *SB* side branch

### Agreement of WSS distribution

**Fig. 2:**
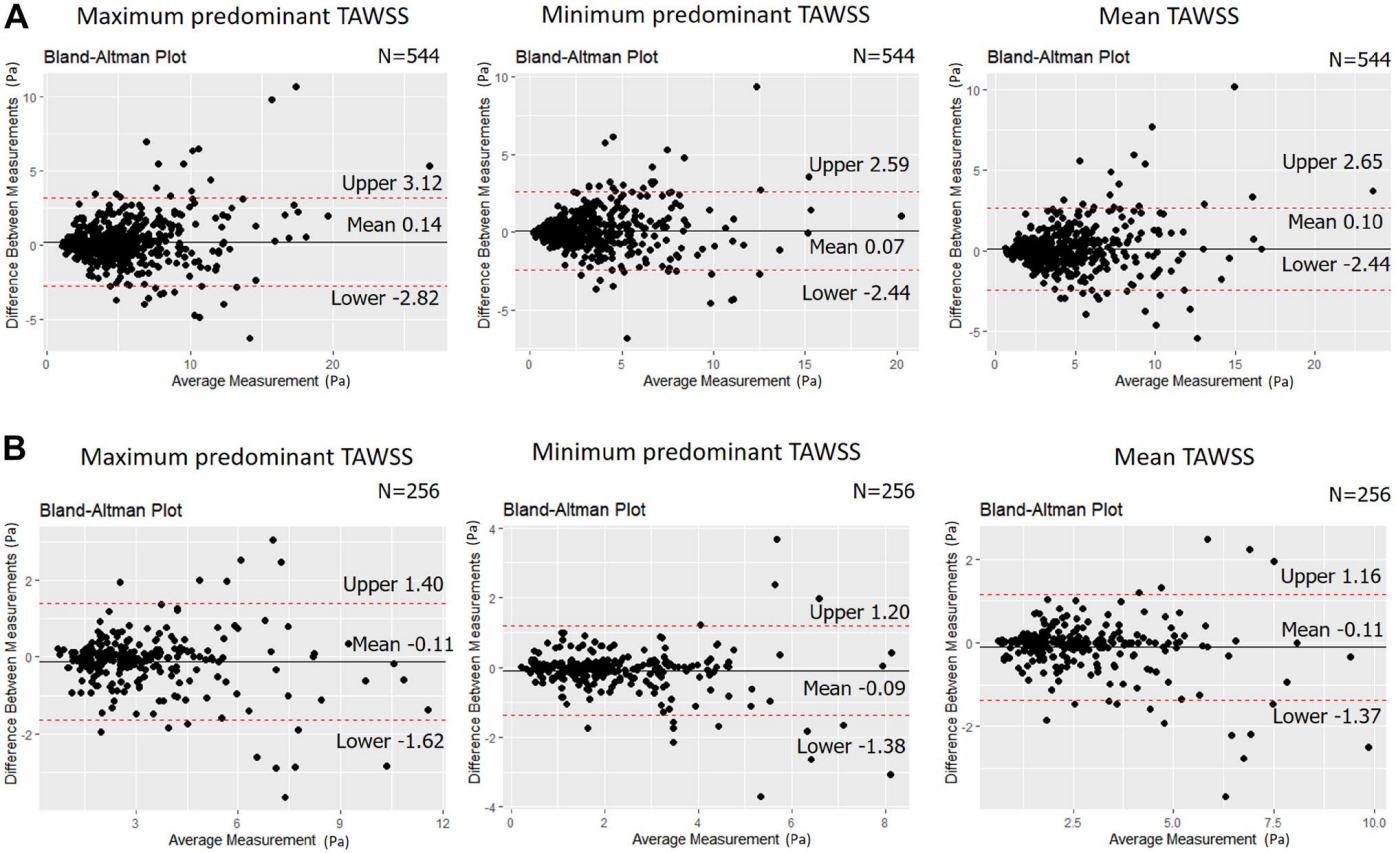
Agreement of two core labs for the maximum predominant TAWSS values 3 mm segments across the entire model in the bifurcated and non-bifurcated models. Bland-Altman analysis for the estimations of the two core labs for the maximum, minimum predominant and mean TAWSS distribution in non-bifurcated (**A**, n = 544) and bifurcated models (**B**, n = 256) for 3mm segments across the entire reconstruction. *TAWSS* time-averaged wall shear stress.

**Fig. 3:**
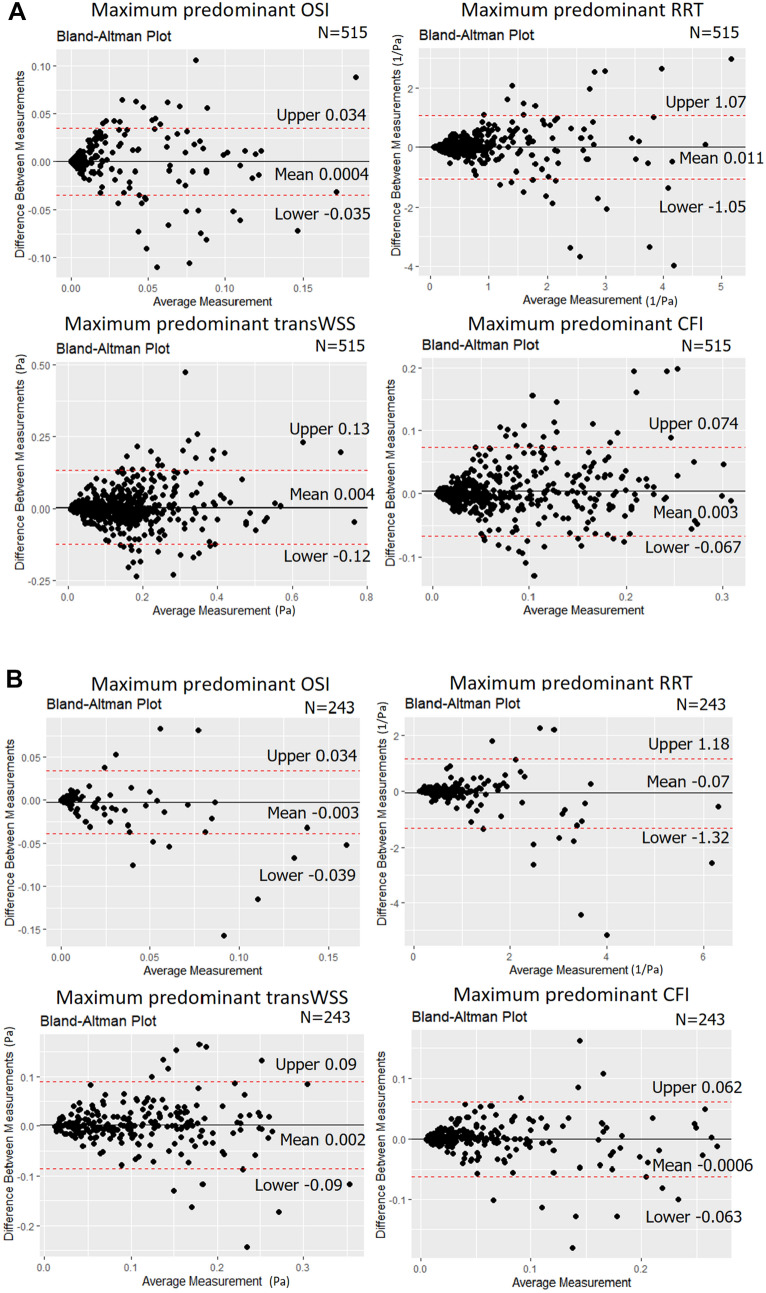
Agreement of two core labs for the maximum predominant multidirectional WSS values 3 mm segments across the entire model in the bifurcated and non-bifurcated models

Mean WSS computation time after 3D model reconstruction with this software was 219 ± 45 s in non-bifurcated and 291 ± 61 s in bifurcated models (processor Intel(R) Core(TM) i9-10900X CPU, 3.70 GHz, RAM 64GB). An excellent ICC of ≥ 0.89 was noted between the two corelabs for the computed TAWSS in the 3 mm segments across the reconstructed model. In bifurcated models, the TAWSS estimations were higher in one corelab than the other but the ICC was again excellent (≥ 0.90; Table 3; Fig. 2). Conversely, the ICC was good-moderate for the multidirectional WSS estimated in 3 mm segments in both non-bifurcated and bifurcated models (ICC range: 0.72–0.86); no significant differences were reported between the estimations of the two corelabs for these metrics in non-parametric comparison (Table 3; Fig. 3).

**Table 3:** Comparison of the WSS metrics in 3 mm segments across the reconstructed models between the two corelabs

	1st corelab estimations	2nd corelab estimations	Mean ± SD of the differences	P value (paired)	ICC(2)	P value (ICC)
*Non-bifurcated models (n = 544)*						
Max. predominant TAWSS (Pa)	5.14 ± 3.54	4.99 ± 3.25	0.14 ± 1.52	0.138	0.90 (0.88–0.92)	< 0.001
Min. predominant TAWSS (Pa)	3.26 ± 2.73	3.19 ± 2.62	0.07 ± 1.28	0.632	0.89 (0.87–0.90)	< 0.001
Mean TAWSS (Pa)	4.16 ± 3.00	4.05 ± 2.81	0.10 ± 1.30	0.311	0.90 (0.88–0.91)	< 0.001
Max predominant OSI	0.012 ± 0.027	0.012 ± 0.028	0.00041 ± 0.0018	0.465	0.80 (0.76–0.83)	< 0.001
Max. predominant RRT (1/Pa)	0.74 ± 0.81	0.73 ± 0.82	0.011 ± 0.54	0.069	0.78 (0.74–0.81)	< 0.001
Max. predominant transWSS (Pa)	0.15 ± 0.12	0.15 ± 0.11	0.0042 ± 0.064	0.629	0.84 (0.81–0.86)	< 0.001
Max. predominant CFI	0.069 ± 0.068	0.066 ± 0.063	0.0033 ± 0.036	0.722	0.85 (0.82–0.87)	< 0.001
*Bifurcated models (n = 256)*						
Max. predominant TAWSS (Pa)	3.23 ± 1.93	3.34 ± 2.05	− 0.11 ± 0.77	0.044	0.92 (0.90–0.94)	< 0.001
Min. predominant TAWSS (Pa)	2.24 ± 1.44	2.33 ± 1.55	− 0.09 ± 0.66	0.053	0.90 (0.88–0.92)	< 0.001
Mean TAWSS (Pa)	2.70 ± 1.57	2.81 ± 1.70	− 0.11 ± 0.64	0.033	0.92 (0.90–0.94)	< 0.001
Max. predominant OSI	0.0081 ± 0.021	0.011 ± 0.029	− 0.0025 ± 0.019	0.499	0.72 (0.66–0.78)	< 0.001
Max. predominant RRT (1/Pa)	0.83 ± 0.79	0.90 ± 1.05	− 0.072 ± 0.64	0.753	0.76 (0.71–0.81)	< 0.001
Max. predominant transWSS (Pa)	0.11 ± 0.070	0.10 ± 0.073	0.0018 ± 0.044	0.37	0.81 (0.76–0.85)	< 0.001
Max. predominant CFI	0.062 ± 0.059	0.062 ± 0.063	− 0.00056 ± 0.032	0.329	0.86 (0.83–0.89)	< 0.001

*WSS* wall shear stress, *TA* time-averaged, *OSI* oscillatory index, *RRT* relative residence time, *transWSS* transverse wall shear stress, *CFI* cross-flow index, *ICC* interclass correlation coefficient, *SD* standard deviation

Comparison of the TAWSS values in 3 mm segments involving the POB showed an excellent ICC for the maximum and minimum predominant TAWSS (0.93 and 0.94, respectively), while a good ICC was reported for the mean TAWSS (0.79, Table 4). For the multidirectional WSS, the ICC ranged between 0.55 and 0.75 except for the maximum predominant OSI where the ICC was weak at 0.44 but statistically not significant (Table 4).

**Table 4:** Comparison of predominant WSS in 3 mm segments involving the point of bifurcation

	1st corelab estimations	2nd corelab estimations	Mean ± SD of the differences	P value (paired)	ICC	P value (ICC)
*Bifurcation lesion (n = 20)*						
Max. predominant TAWSS (Pa)	3.44 ± 1.93	3.39 ± 1.99	0.055 ± 0.68	1	0.94 (0.87–0.98)	< 0.001
Min. predominant TAWSS (Pa)	2.29 ± 1.61	2.09 ± 1.33	0.20 ± 0.97	0.288	0.79 (0.57–0.91)	< 0.001
Mean TAWSS (Pa)	2.76 ± 1.70	2.67 ± 1.52	0.093 ± 0.64	0.393	0.93 (0.83–0.97)	< 0.001
Max. predominant OSI	0.13 ± 0.25	0.15 ± 0.24	− 0.0015 ± 0.027	0.898	0.44 (0.01–0.73)	0.024
Max. predominant RRT (1/Pa)	1.08 ± 1.00	1.16 ± 1.11	− 0.081 ± 0.831	0.546	0.70 (0.39–0.87)	< 0.001
Max. predominant transWSS (Pa)	0.11 ± 0.04	0.12 ± 0.05	− 0.005 ± 0.040	0.898	0.55 (0.16–0.79)	0.005
Max. predominant CFI	0.08 ± 0.06	0.08 ± 0.06	− 0.003 ± 0.043	0.277	0.75 (0.48–0.89)	< 0.001

*WSS* wall shear stress, *TA* time-averaged, *OSI* oscillatory index, *RRT* relative residence time, *transWSS* transverse wall shear stress, *CFI* cross-flow index, *ICC* interclass correlation coefficient, *SD* standard deviation

**Fig. 4:**
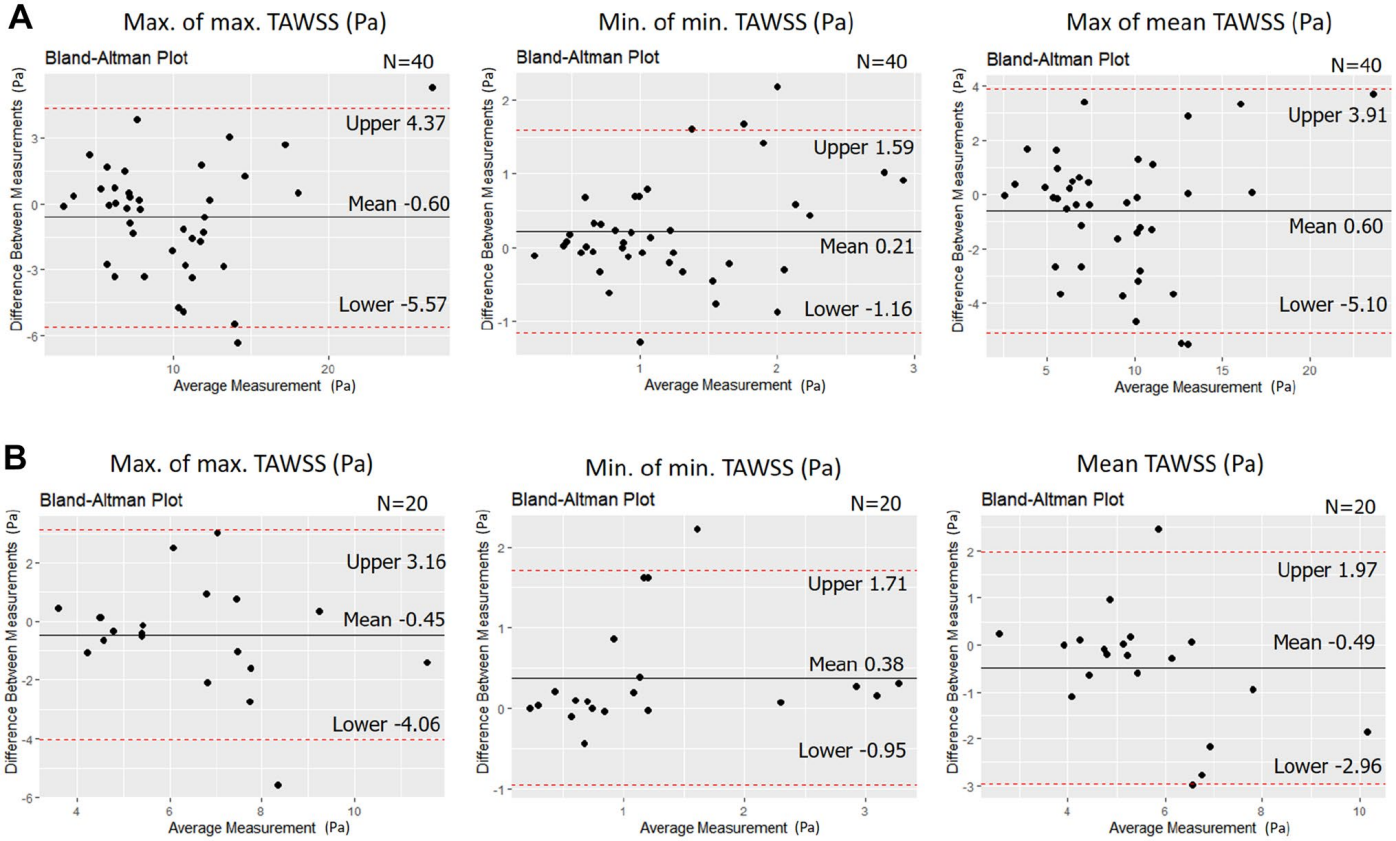
Agreement of the maximum of maximum, minimum of minimum predominant and maximum of mean TAWSS distribution in the bifurcated and non-bifurcated models at a lesion level. Comparison of TAWSS components in Bland-Altman Plot; A: straight models (n=40) and B: bifurcation models (n=20). TAWSS, time-averaged wall shear stress.

Finally, lesion level analysis demonstrated a moderate ICC 0.53–0.85 for the lesion maximum, maximum predominant and minimum predominant TAWSS, while non-parametric test showed a significant inter-corelab difference for the minimum predominant TAWSS in bifurcation lesions (Table 5). The ICC was moderate for the maximum predominant transWSS and RRT (range: 0.60–0.74) while for the maximum predominant OSI and CFI, the ICC was smaller in bifurcated models than the non-bifurcated reconstructions (Table 5; Fig. 4).

**Table 5:** Comparison of the WSS metrics between two Corelabs at a lesion level

	1st corelab estimations	2nd corelab estimations	Mean ± SD of the differences	P value (paired)	ICC	P value (ICC)
*Straight lesion (n = 40)*						
Max. predomiant TAWSS (Pa)	9.52 ± 4.84	10.11 ± 4.60	− 0.60 ± 2.54	0.216	0.85 (0.74–0.92)	< 0.001
Min predomiant TAWSS (Pa)	1.31 ± 0.83	1.10 ± 0.62	0.21 ± 0.70	0.128	0.53 (0.27–0.72)	< 0.001
Max. TAWSS (Pa)	8.62 ± 4.31	9.22 ± 4.18	− 0.60 ± 2.30	0.202	0.85 (0.73–0.92)	< 0.001
Max. predominant OSI	0.054 ± 0.052	0.058 ± 0.051	− 0.0041 ± 0.037	0.745	0.74 (0.57–0.86)	< 0.001
Max. predominant RRT (1/Pa)	1.90 ± 1.30	2.08 ± 1.37	− 0.18 ± 1.19	0.64	0.60 (0.36–0.76)	< 0.001
Max. predominant transWSS (Pa)	0.23 ± 0.10	0.22 ± 0.09	0.012 ± 0.084	0.818	0.63 (0.40–0.78)	< 0.001
Max. predominant CFI	0.16 ± 0.08	0.16 ± 0.06	− 0.005 ± 0.064	0.368	0.62 (0.39–0.78)	< 0.001
*Bifurcation lesion (n = 20)*						
Max. predominant TAWSS (Pa)	6.21 ± 1.93	6.66 ± 2.40	− 0.45 ± 1.84	0.261	0.64 (0.30–0.84)	< 0.001
Min. predominant TAWSS (Pa)	1.44 ± 1.03	1.06 ± 0.94	0.38 ± 0.68	0.004	0.72 (0.37–0.88)	< 0.001
Max. TAWSS (Pa)	5.33 ± 1.46	5.82 ± 2.00	− 0.46 ± 1.26	0.076	0.72 (0.43–0.88)	< 0.001
Max. predominant OSI	0.032 ± 0.043	0.053 ± 0.064	− 0.021 ± 0.057	0.105	0.43 (0.03–0.72)	0.020
Max. predominant RRT (1/Pa)	1.84 ± 1.57	2.54 ± 2.13	− 0.69 ± 1.61	0.112	0.60 (0.24–0.82)	0.001
Max. predominant transWSS (Pa)	0.17 ± 0.08	0.20 ± 0.08	− 0.024 ± 0.061	0.153	0.69 (0.37–0.86)	< 0.001
Max. predominant CFI	0.12 ± 0.08	0.15 ± 0.08	− 0.030 ± 0.084	0.095	0.42 (0.012–0.72)	0.024

*WSS* wall shear stress, *TA* time-averaged, *OSI* oscillatory index, *RRT* relative residence time, *transWSS* transverse wall shear stress, *CFI* cross-flow index, *ICC* interclass correlation coefficient, *SD* standard deviation

### Agreement of MACE-related factors

**Fig. 5:**
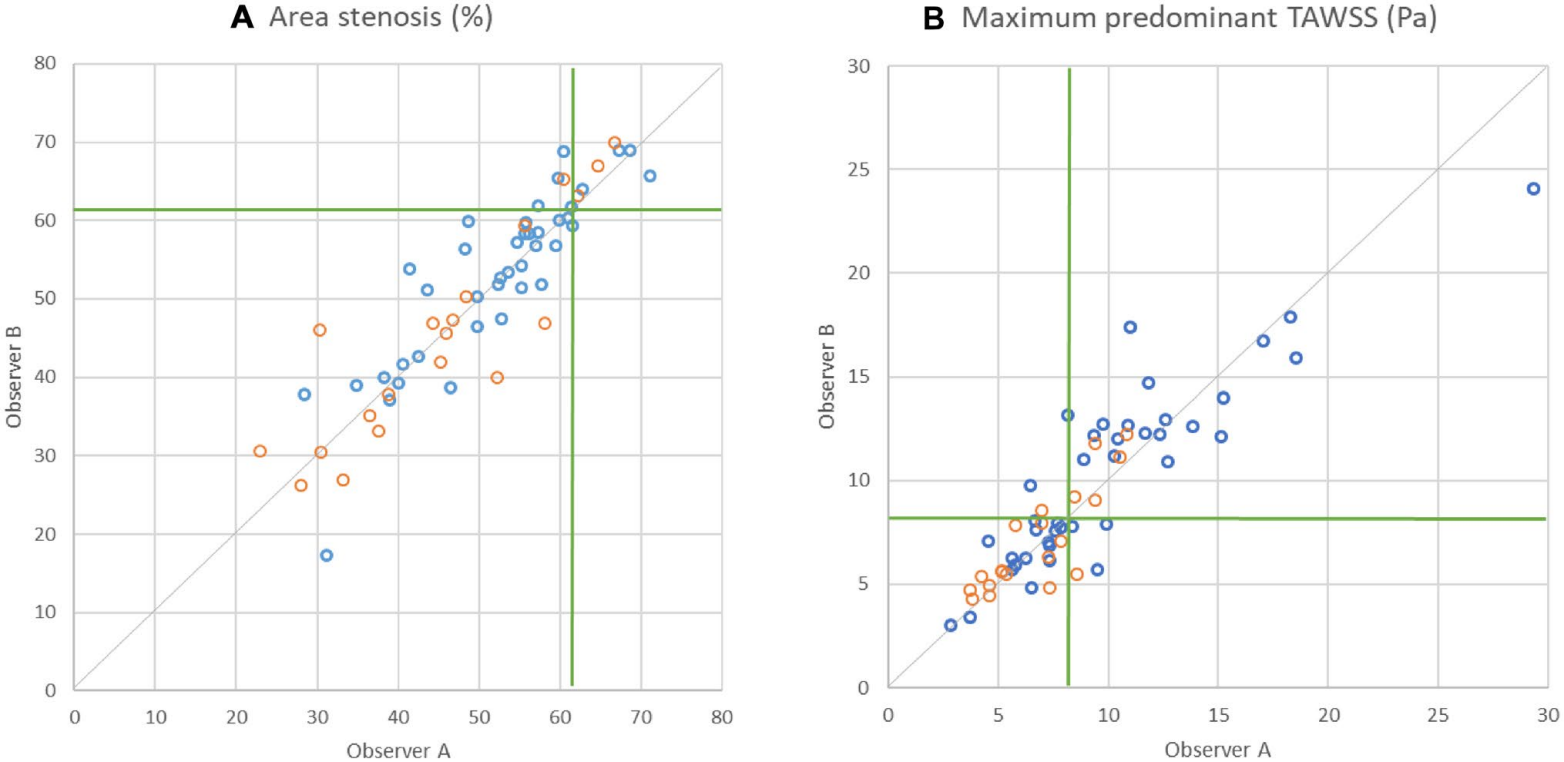
Correlation between 2 corelabs concerning area stenosis and maximum predominant TAWSS Scatter plots of area stenosis (A) and maximum predominant TAWSS in the lesion (B) were examined by Corelabs A and B. Blue plots came from straight models and orange plots from bifurcation models. The Green line shows the optimal threshold to predict major adverse cardiac events. Abbreviations: TAWSS, time-averaged wall shear stress.

When using the cut-off value of maximum TAWSS and % AS to define high-risk lesions a substantial agreement was noted between the two core labs; a κ of 0.77 was reported for detecting lesions with a maximum TAWSS > 8.24 Pa and a κ of 0.71 for detecting lesions with AS > 61.3% (Fig. 5).

## Discussion

This is the first study that examined the inter-corelab variability of the CAAS Workstation WSS software. We found an excellent agreement for the 3D-QCA and the mean and predominant TAWSS estimations in 3 mm segments in both bifurcated and non-bifurcated models while the ICC between the two corelabs for the multidirectional WSS estimations ranged from weak to moderate. More importantly, the two corelabs had a high agreement in identifying lesions with a high-risk morphology that are exposed to an unfavourable haemodynamic environment that is likely to progress and cause events.

Two studies have assessed the efficacy of the CAAS Workstation WSS software in measuring the WSS distribution against conventional CFD analyses and reported an excellent agreement between the estimations of the conventional approach and the CASS software. These studies also assess the inter- and intra-obsever variability of the CAAS Workstation WSS software reporting excellent reproducibility for the computed WSS. The present analysis provides additional insights into the reproducibility of this novel software reporting for the first time the agreement of two corelabs for the 3D-QCA estimations and WSS metrics.

The agreement of the two experts for the 3D-QCA estimations was excellent despite the fact the QCA analysis was not fully automated and relied on the two analysts who made corrections of the lumen borders when these were deemed necessary. The high reproducibility of the two corelabs in the 3D-QCA estimations affected the inter-corelab reproducibility for the WSS metrics. We found a high ICC between the estimations of the two corelabs for the mean and predominant TAWSS estimated in 3 mm segments across the entire model. The inter-corelab agreement was good- however when analysis focused on the POB where disturbed flow patterns are expected, while lesion level analysis demonstrate a moderate ICC and narrow limits of agreement for the TAWSS Despite the ICC of 0.54 for the minimum predominant TAWSS in the non-bifurcated models, the bias was small and the limits of agreement were narrow for this variable. Conversely, the ICC was excellent for the 3D-QCA analysis. More importantly, the agreement of the two corelabs was high in detecting lesions with a high-risk morphology exposed to high TAWSS that as previous reports have shown are likely to progress and cause events. These findings indicate that the estimations of the CAAS Workstation WSS software are reproducible, and underscore its potential clinical value for more accurate risk stratification and vulnerable plaque detection.

In contrast to the study of Candreva et al. and Tufaro et al. our study evaluated for the first time the agreement of the two corelabs in the computation of the TAWSS and multidirectional WSS distribution. We found slightly different OSI, RRT, transWSS and CFI values compared to previous reports which however used intravascular ultrasound (IVUS) and X-ray angiography to reconstruct vessel anatomy. [11, 18] It is likely that the incorporation of the IVUS data in the 3D models to allow more precise reconstruction of lumen geometry including the minor irregularities of the lumen surface that cannot be captured by 3D-QCA which assumes that luminal cross-sections have a circular or ellipsoid morphology. The latter approximation may affect the multi-directional WSS computation resulting in current values as these are heavily dependent on model architecture. These findings highlight the need to conduct studies that will explore the value of 3D-QCA-based modelling in measuring multidirectional WSS using intracoronary imaging based-reconstruction as a reference standard. [18, 22]

We also found a variable agreement between the two corelabs for the multidirectional WSS metrics. For the entire model, the ICC ranged from 0.72 to 0.86 in bifurcated and non-bifurcated models indicating a moderate-good agreement between the two corelabs. Conversely, when analysis focused on the POB the ICC was low for the OSI and tranWSS suggesting a weak inter-corelab reproducibility for these metrics in this region. This can be explained by the fact that minor changes in the delineation of the main and side branch and the fusion of these branches may have a detrimental effect on the POB geometry and OSI and transWSS values. Similarly, we found a weak to moderate agreement of the two corelabs for the multidirectional WSS at the lesion site—a finding that highlights the limited reproducibility of the software for these metrics in diseased segments. Of note, it has to be stressed that this is the first study that assessed the reproducibility of multidirectional WSS computation in coronary reconstructions as previous intravascular imaging-based CFD analyses have not tested the inter- and intra-observer agreement for these metrics. Our findings underscore the need to examine the reproducibility of multidirectional WSS computations in intravascular imaging-based reconstructions and explore their added value in plaque progression over TAWSS.

In this study, WSS computation time was less than 4 min for non-bifurcated and less than 5 min for bifurcated models including 3D-QCA reconstruction. These findings are in line with previous reports [7] supporting the potential of this software for clinical use. However, before advocating the use of CAAS Workstation WSS software in clinical practice further research is needed to explore in retrospective but mainly in prospective studies its value in detecting high-risk lesions and patients.

### Limitation

Several limitations of the present analysis should be acknowledged. Firstly, although in total 60 lesions were included in the analysis, the number of bifurcated lesions was relatively small. Secondly, this is a retrospective analysis of already acquired data and although we excluded angiographic cases with poor image quality it is likely prospective angiographic data to have superior image quality and their analysis to be associated with a higher inter-corelab agreement. Third, lesion level analysis was performed in borderline non-flow limiting lesions; it is unclear whether our findings also apply to lesions with mild or severe stenosis. Finally, the present study did not take into account the non-Newtonian behaviour of the blood which can affect flow patterns in severely stenotic lesions [23]; this simplification however enabled fast WSS computation rendering this analysis clinically feasible.

## Conclusions

The CAAS workstation enables fast and reproducible 3D-QCA reconstruction and computation of WSS metrics in both bifurcated and non-bifurcated segments. These features are essential for clinical applications, however, further research is needed to explore their efficacy in predicting cardiovascular events before considering their use in the clinical setting.

## Supplementary information

Below is the link to the electronic supplementary material.

Supplementary material 1 (DOCX 622.1 kb)
