# Supplementary material for: Agreement of wall shear stress distribution between two core laboratories using three-dimensional quantitative coronary angiography
Source: Int J Cardiovasc Imaging. 2023 May 27;39(8):1581–92. doi: 10.1007/s10554-023-02872-4 (PMC10427706; doi:10.1007/s10554-023-02872-4)
Supplement: Supplementary file 1 — Supplementary material 1 (DOCX 622.1 kb) [file 10554_2023_2872_MOESM1_ESM.docx]

**Agreement of wall shear stress distribution between two core laboratories using three-dimensional quantitative coronary angiography**

**Short title:** Inter Corelab agreement of wall shear stress

Shigetaka Kageyama, MD^1^,# Vincenzo Tufaro, MD^2,3,4^,# Ryo Torii, PhD^5^, Grigoris Karamasis, MD^6^, Roby D Rakhit, MD^7,12^, Eric K. W. Poon, PhD^8^, Jean-Paul Aben, MSc^9^, Andreas Baumbach, MD, PhD^2,3,10^, Patrick W. Serruys, MD, PhD^1,11^, Yoshinobu Onuma, MD, PhD^1^, Christos V. Bourantas, MD, PhD^2,3,12^

#Equally contributed to the writing of the manuscript

**Supplemental materials**

**Table 1s.** Overview and mathematical description of the shear stress metrics

| Shear stress metric | Description | Mathematical formula |
| --- | --- | --- |
| Time-averaged WSS (TAWSS), Pa | Shear stress averaged over the cardiac cycle | $\frac{1}{T}\int_{0}^{T} \left\vert\tau_{w}(x,t) \right\vert dt$ |
| Oscillatory shear index (OSI) | Ratio between backward and forward going shear stress | $0.5\left( 1-\frac{\left\vert\int_{0}^{T} \tau_{w}(x,t)dt \right\vert}{\int_{0}^{T} \left\vert\tau_{w}(x,t) \right\vert dt} \right)$ |
| Relative residence time (RRT), Pa^-1^ | Relative time that a blood particle resides at a certain location at the vessel wall | $\frac{1}{\mathrm{TAWSS}(x)\left\{ 1-2 OSI\left( x \right) \right\}}$ |
| Transverse WSS (transWSS), Pa | Shear stress vector in perpendicular direction to the main flow direction | $\frac{1}{T}\int_{0}^{T} \left\vert\tau_{w}(x,t)\cdot p(x) \right\vert dt$ |
| Cross-flow index (CFI) | The transWSS normalized for the time-averaged wall shear stress | $\frac{1}{T}\int_{0}^{T} \left\vert\frac{\tau_{w}(x,t)}{\left\vert\tau_{w}(x,t) \right\vert}\cdot p(x) \right\vert dt$ |

(𝑥,𝑡) is the instantaneous shear stress vector for each node 𝑥 and time 𝑡. The cardiac cycle time is T. 𝑝(𝑥) is the vector indicating the cross flow direction which is perpendicular to both the surface normal 𝑛(𝑥) and the mean wall shear stress vector. Abbreviation: WSS, wall shear stress.

**Figure 1s.** Intercolab variability of 3D-QCA analysis metrics in bifurcated and non-bifurcated modes.


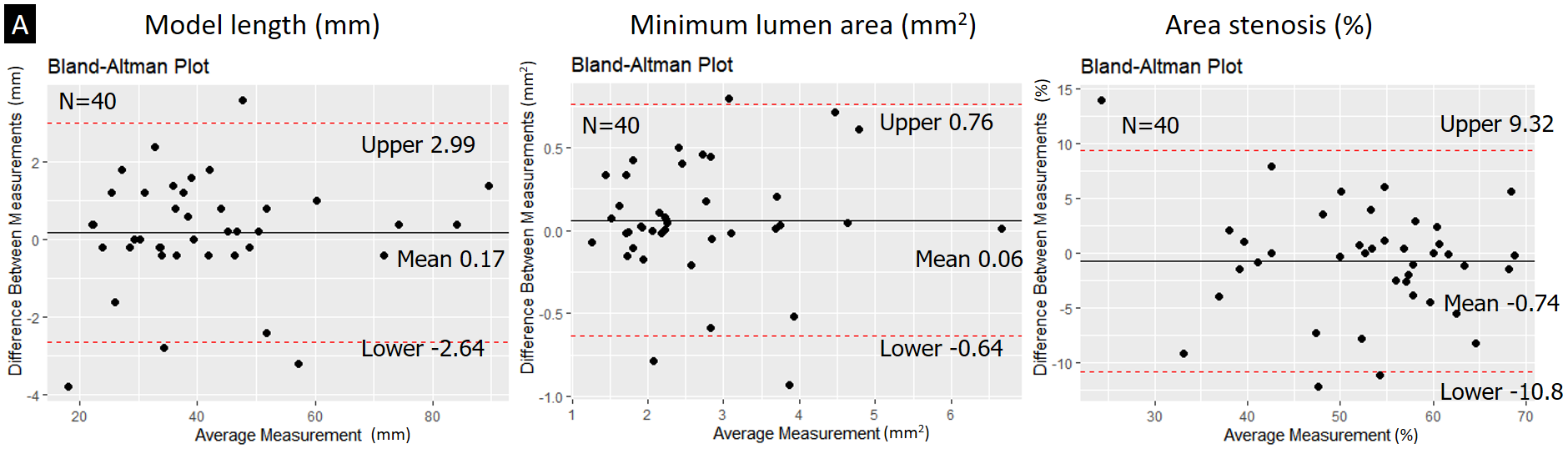

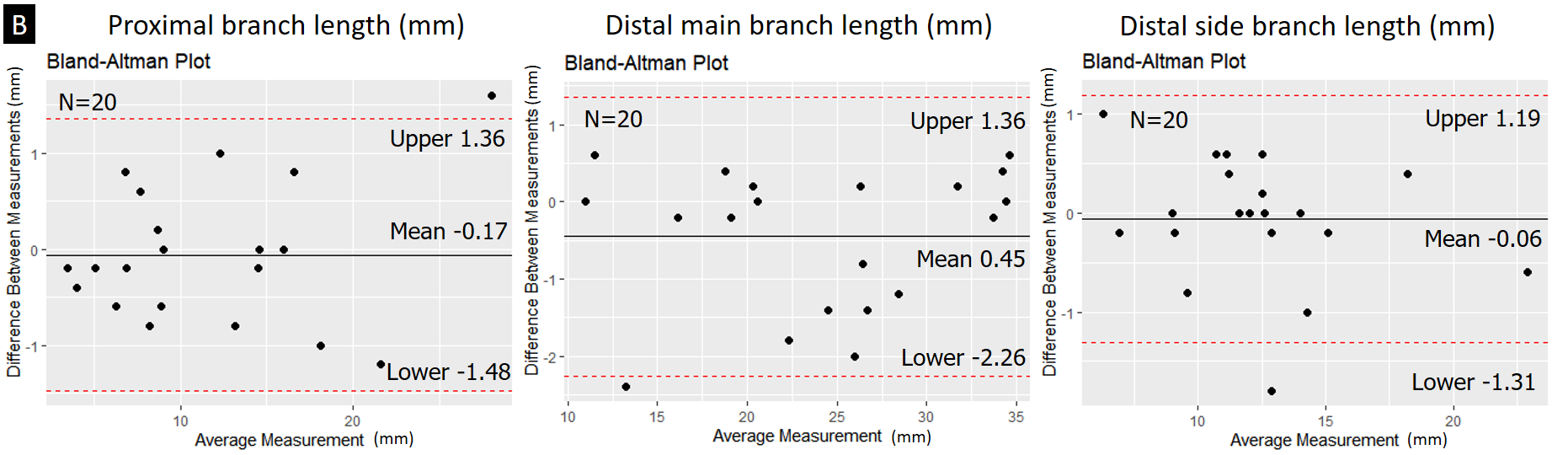

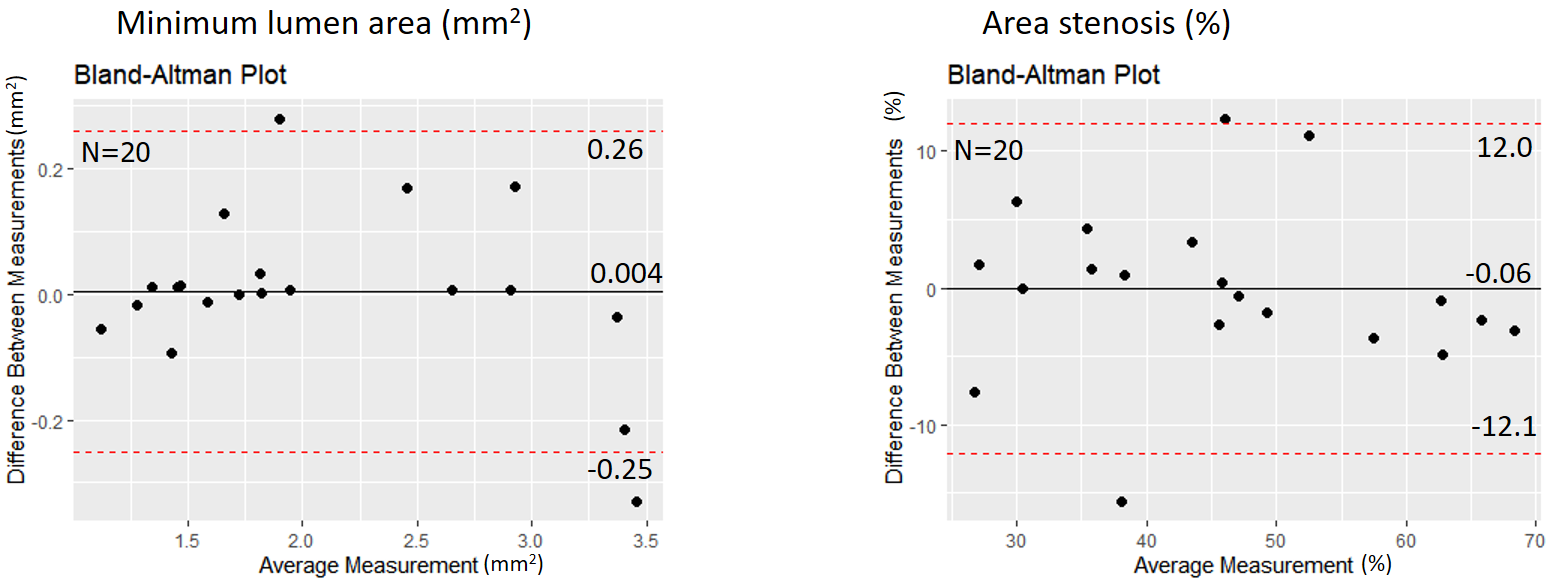


Comparison of 3D-QCA components in Bland-Altman Plot; A: straight models (n=40) and B: bifurcation models (n=20).

Abbreviations: 3D, three-dimensional; QCA, quantitative coronary angiography.
